# Tau in Alzheimer's disease: Shaping the future patient journey

**DOI:** 10.1016/j.tjpad.2025.100447

**Published:** 2026-01-01

**Authors:** Catherine J. Mummery, Christopher Chen Li-Hsian, Cristian A. Lasagna-Reeves, Rik Ossenkoppele, Christopher C. Rowe, Douglas W. Scharre, Huali Wang, Simon Kyaga, Jeffrey L. Cummings

**Affiliations:** a Dementia Research Centre, Institute of Neurology, University College London, Queen Square, London, UK; b Memory Aging and Cognition Centre, Department of Pharmacology, Yong Loo Lin School of Medicine, National University of Singapore, Singapore, Singapore; c Department of Neurology, Baylor College of Medicine, Houston, TX, USA; d Clinical Memory Research Unit, Department of Clinical Sciences Malmö, Faculty of Medicine, Lund University, Lund, Sweden; e Alzheimer Center Amsterdam, Neurology, Vrije Universiteit Amsterdam, Amsterdam UMC Location VUmc, Amsterdam, the Netherlands; f Amsterdam Neuroscience, Neurodegeneration, Amsterdam, the Netherlands; g Department of Molecular Imaging and Therapy, Austin Health, Melbourne, Victoria, Australia; h The Florey Institute of Neuroscience and Mental Health, The University of Melbourne, Melbourne, Victoria, Australia; i Division of Cognitive Neurology, Department of Neurology, The Ohio State University Wexner Medical Center, Columbus, OH, USA; j Dementia Care and Research Center, Peking University Institute of Mental Health (Sixth Hospital), Beijing, China; k Biogen International GmbH, Neuhofstrasse, Baar, Switzerland; l Chambers-Grundy Center for Transformative Neuroscience, Department of Brain Health, Kirk Kerkorian School of Medicine, University of Nevada Las Vegas, Las Vegas, NV, USA

**Keywords:** Alzheimer’s disease, Tau, Biomarkers, Clinical pathways

## Abstract

Alzheimer’s disease is a complex and multifactorial disease characterized by two key pathological hallmarks: amyloid-beta plaques and tau neurofibrillary tangles. Recent progress has led to the development and approval of disease-targeted therapies for Alzheimer’s disease in the form of anti-amyloid-beta monoclonal antibodies. However, findings suggest that amelioration of multiple pathological drivers may be required to maximize clinical effect. An increasing body of evidence suggests that tau is a critical player in Alzheimer’s disease pathophysiology, contributing significantly to neurodegeneration and cognitive decline. There are now several tau-targeting drugs in clinical development. In this review, we build on research and advancements in the field of tau to envision how an increasing focus on tau could shape the future Alzheimer’s disease patient journey. We highlight the potential of tau as both a promising therapeutic target and a valuable biomarker, with the potential to inform treatment decisions and provide insight into disease trajectories. We also consider what a greater focus on tau may bring to an already evolving patient care pathway characterized by an increased influx of patients presenting earlier in the disease continuum, changes in workflow and infrastructural requirements, and increased complexity in treatment decision-making, treatment administration, treatment monitoring, and patient tracking. This review underscores the critical changes that may be required and knowledge gaps to be elucidated to ensure healthcare system preparedness for additional classes of disease-targeted therapy to move toward a next-generation, individualized treatment approach to Alzheimer’s disease diagnosis and care.

## 1 Introduction

Alzheimer’s disease (AD) is a chronic, progressive, neurodegenerative disease, and the most common cause of dementia, accounting for up to 60–80 % of cases of late-onset cognitive decline [1]. The pathophysiology of AD is complex and multifactorial, with two types of protein aggregates, amyloid-beta (Aβ) plaques and tau neurofibrillary tangles (NFTs), as the key pathological hallmarks [2]. These pathogenic markers begin to accumulate in the preclinical stage of the AD continuum before symptoms of cognitive decline are evident. The disease progresses clinically to mild cognitive impairment (MCI) and finally to dementia, where symptoms interfere with daily function and eventually result in a complete loss of independence and death. In addition to protein aggregation, AD is characterized by synaptic dysfunction, inflammation, brain metabolic abnormalities, and neuronal degeneration [3]. It is estimated that 416 million people globally have AD pathology (Aβ-positive), with 32 million of those having AD dementia [4]. Despite the enormous burden of AD, on both an individual and societal level [5], there was an almost two-decade hiatus where no new AD therapies emerged. However, in the past 5 years, the AD treatment landscape has experienced a transformation, with parallel breakthroughs in both treatments and diagnostic techniques [[6], [7], [8], [9], [10]]. Prior to this, the only treatment options available to patients with AD were symptomatic (i.e., did not interrupt the cascade that characterizes the disease pathophysiology) [11]. Recent progress has now led to the development and approval of disease-targeted therapies (DTTs) for AD – providing, for the first time, treatment options with the potential to change the disease course. Two anti-Aβ monoclonal antibodies – lecanemab, and donanemab – have received full approval by the US Food and Drug Administration (FDA) and other regulatory agencies based on their ability to slow cognitive decline in early-stage symptomatic AD [8,9].

The early focus of DTT development has been on amyloid targets [12]. The emergence of anti-Aβ monoclonal antibodies represents an important turning point in AD drug development; however, despite demonstrating effective Aβ-plaque clearance and slowing of clinical decline in patients with early symptomatic AD, cognition and function continue to worsen over time [[7], [8], [9]]. This suggests that amelioration of other pathological targets is needed to further slow the clinical decline. Investigational drugs targeting a number of these potential pathological targets are currently in clinical development [12].

Of the key pathological characteristics of AD, Aβ plaque and related Aβ change are the first measurable markers of disease detectable in the neocortex. However, there is an increasing body of evidence that suggests tau is a critical player in the pathophysiology of AD. Neocortical tau tangles accumulate later in the disease course, closer to when clinical symptoms occur, and have been found to correlate more strongly with the severity and progression of symptomatology than Aβ plaque deposition [6,13]. Moreover, biomarkers representing the level of neocortical tau tangles in the preclinical and MCI stages of AD have demonstrated promising prognostic potential, showing a stronger association with future cognitive decline than Aβ plaque deposition or magnetic resonance imaging (MRI) markers [14,15]. Tau protein NFTs imaged by tau positron emission tomography (PET) at treatment initiation have been shown to impact the magnitude of treatment response with anti-Aβ monoclonal antibodies [8]. Taken together, tau is a promising therapeutic target and potentially valuable prognostic and pharmacodynamic biomarker.

In this review, we provide analysis and perspectives on research and advancements in the field of tau, ranging from the role of tau biomarkers in the diagnosis and staging of AD to the evolving landscape of tau as a therapeutic target, and envision how a greater focus on tau could shape the future AD patient journey.

## 2 The role of tau in AD pathophysiology

In healthy physiological conditions, tau is an unfolded, highly soluble microtubule-associated protein that is essential for normal neuronal functioning [[16], [17], [18]]. Tau is encoded by the microtubule-associated protein tau (*MAPT)* gene, and through alternative splicing, this gene produces six isoforms of tau protein in the brain. These isoforms can be differentiated by the presence of zero, one, or two N-terminal inserts, and the presence of either three (3R) or four (4R) microtubule-binding domain repeats [19]. The resulting translated tau ranges from 352 to 441 amino acids in length [16]. In the cerebral cortex of healthy adults, 3R and 4R tau isoforms are present in approximately equal amounts [20]. Disruption of the ratio of 3R-tau and 4R-tau isoforms has been implicated in AD, with the 4R-tau isoform thought to have a greater propensity to aggregate and form NFTs [21].

Once tau has been translated, post-translational modifications (PTMs) are essential to the normal function of the protein. However, under pathological states, such as in AD, tau can undergo abnormal PTMs, leading to protein dysfunction, accumulation, and aggregation [22]. Aberrant PTMs and tau aggregation are not unique to AD – misfolded tau aggregates are characteristic of numerous age-related neurodegenerative diseases, termed tauopathies. Tauopathies can be categorized as primary or secondary. In primary tauopathies, such as progressive supranuclear palsy and Pick’s disease, tau is the predominant protein abnormality. In secondary tauopathies, tau protein aggregates coexist with other protein abnormalities [23]. AD is in the latter category, in which Aβ and tau proteins are synergistically linked within the pathological process [24]. Tauopathies can be further categorized by the predominant tau isoforms involved, resulting in 3R, 4R, or mixed 3R/4R tauopathies. Progressive supranuclear palsy is a 4R tauopathy, Pick’s disease is a 3R tauopathy, and AD is a 3R/4R tauopathy [23,25].

**Fig. 1:**
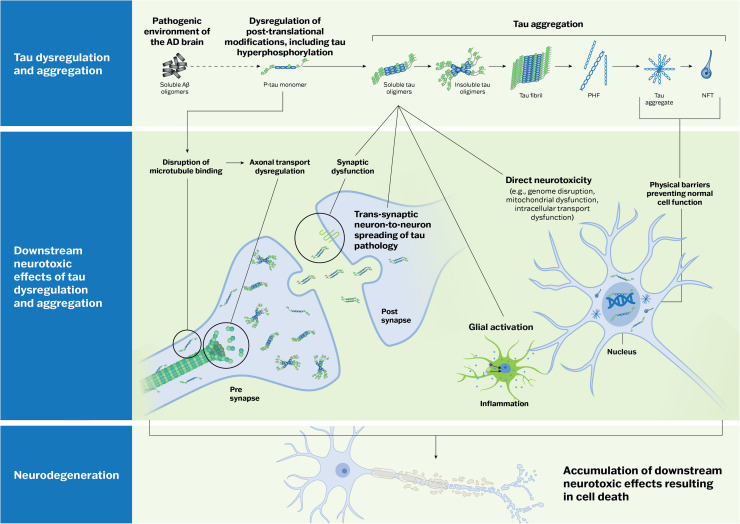
The role of tau in Alzheimer’s disease pathophysiology. In the pathogenic environment of the AD brain, the equilibrium between tau kinase and phosphatase activities can become disrupted, leading to tau hyperphosphorylation. This causes dissociation of tau from microtubules and axonal transport dysregulation, leading to synaptic dysfunction and neurodegeneration. Hyperphosphorylated tau monomers self-aggregate to form dimers, followed by further aggregation into oligomers and NFTs. Tau oligomers directly exhibit neurotoxicity (e.g., via genome destruction, disruption of mitochondrial function, impairment of cell signaling, synaptic dysfunction, and impairment of the protein degradation system). They also drive a potent glial response, contributing to neuroinflammation. Large tau aggregates and NFTs cause direct neurotoxicity, acting as physical barriers in the cytoplasm and thus, compromising normal cell metabolism. The neuron-to-neuron spread of pathological intracellular tau is thought to occur through a “prion-like” seeding mechanism, whereby oligomeric tau is secreted and transferred to a recipient neuron transsynaptically, where it then recruits endogenous tau and transmits its misfolding properties. Abbreviations: Aβ, amyloid beta; AD, Alzheimer’s disease; NFT, neurofibrillary tangle; p-tau, phosphorylated tau; PHF, paired helical filaments.

Tau phosphorylation is the most studied PTM in AD pathophysiology; it has a key influence on both the regulation of normal tau function and driving AD pathological processes. The phosphorylation of tau is essential to normal neuronal functioning by regulating tau’s affinity to microtubules, which in turn mediates microtubule assembly and stabilization [26,27]. This process is highly regulated by a balance between tau kinase and phosphatase activities. In the pathogenic environment of the AD brain, this equilibrium can become disrupted, leading to tau hyperphosphorylation [[28], [29], [30]]. Phosphorylation of tau at amino acid residues responsible for microtubule binding can cause dysregulation of axonal transport [[31], [32], [33]], leading to synaptic dysfunction and neurodegeneration [[34], [35], [36]]. Phosphorylation of tau at amino acid residues outside of those responsible for microtubule binding can lead to tau aggregation [37]. Hyperphosphorylated tau monomers self-aggregate to form dimers, followed by further aggregation into oligomers, pre-tangles, and NFTs (Fig. 1) [[38], [39], [40]]. Tau oligomers have been shown to exhibit neurotoxicity via numerous pathways, including genome destruction, disruption of mitochondrial function, impairment of cell signaling, synaptic dysfunction, and impairment of protein degradation [41]. Oligomers also drive a potent glial response, contributing to neuroinflammation [42,43]. Oligomeric tau can fibrilize to form paired helical filaments (PHFs) or tightly wrapped straight filaments, and ultimately to NFTs. PHFs are the primary component of NFTs in AD; straight filaments are less abundant [[38], [39], [40]]. NFTs can cause direct neurotoxicity, acting as physical barriers in the cytoplasm and compromising normal cell metabolism [44].

Although phosphorylation has been the most studied PTM of tau to date, there is increasing evidence showing that many other PTMs play a significant role in the regulation of tau [22] and can become dysregulated in pathological conditions [[45], [46], [47], [48]]. These alternative PTMs include acetylation, ubiquitination, glycation, glycosylation, methylation, or truncation. Tau PTM is a rapidly evolving area of study and is the target of numerous investigational tau-directed therapies [49,50].

The spreading of tau pathology follows a predictable pattern in AD, which can be visualized and staged using tau PET [51,52]. Hyperphosphorylated tau aggregates first accumulate in the brainstem, including the locus coeruleus, before progressing to the cerebral cortex, with the earliest cortical involvement typically seen in the transentorhinal and entorhinal regions of the medial temporal lobe (MTL; Braak stages I/II). Tau aggregates then spread through synaptically connected neural circuits to the limbic regions (Braak stages III/IV) and finally progress to moderate-to-severe neocortical involvement (Braak stages V/VI) [[53], [54], [55]]. The neuron-to-neuron spread of pathological intracellular tau is thought to occur through a prion or “prion-like” seeding mechanism, whereby oligomeric tau is secreted and transferred to a recipient neuron transsynaptically, where it then recruits endogenous tau and transmits its misfolding properties [[56], [57], [58]]. This progressive topographical spread of NFT pathology has shown a strong correlation with synaptic loss, neurodegeneration, and cognitive decline (Fig. 1) [[59], [60], [61], [62], [63]].

## 3 Tau as a therapeutic target in the future AD treatment landscape

**Fig. 2:**
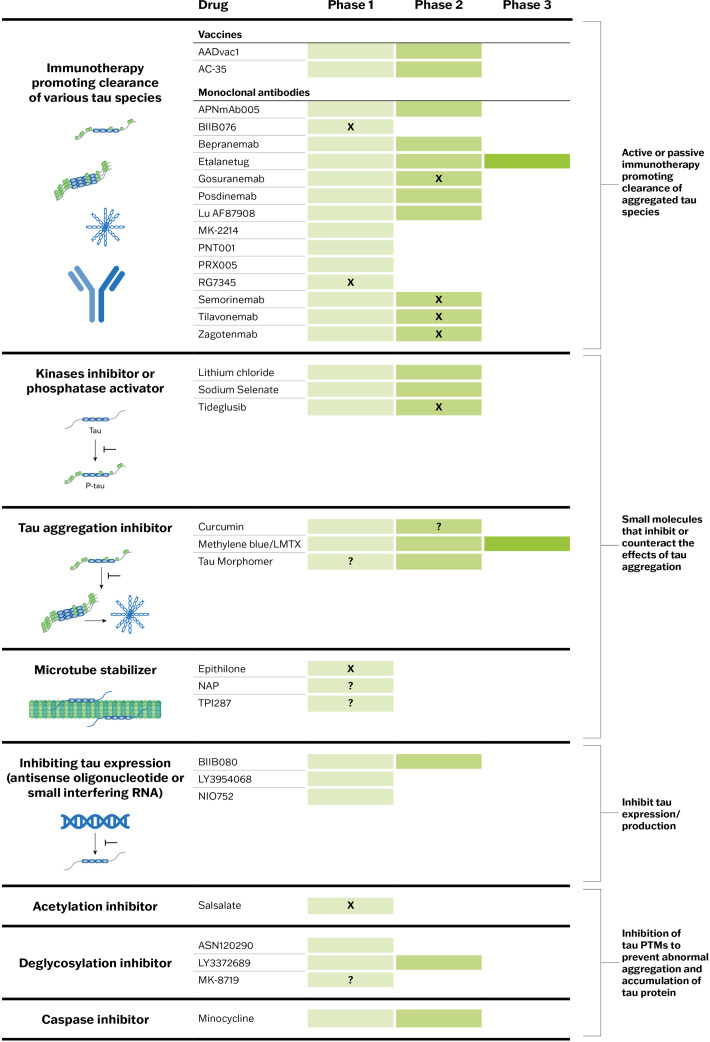
Target engagement of tau-directing therapies in clinical trials to date. Clinical trial status of tau-directed therapies, by target engagement, at time of writing. “X” indicates the development of the drug has been terminated, and “?” indicates the current development status is uncertain. Abbreviations: PTM, post-translational modification.

There is a strong rationale for the investigation of tau as a therapeutic target for AD [13]. Tau suppression in AD may reduce tau spreading and aggregation and reduce Aβ-related toxicity. Several tau-directed therapies, each with differing modes of action, are in various stages of clinical development (Fig. 2) [49]. Therapeutic strategies include inhibiting abnormal tau production, preventing misfolding and aggregation of tau, and blocking tau spread between neurons (tau propagation). The clinical efficacy of tau-directed therapies has yet to be established, and several trials, primarily of tau-directed immunotherapies, have been negative. To date, failures have been attributed to several factors, including: (1) choice of epitope, (2) study population, and (3) mechanism of action [64]. This reinforces the importance of developing the “right drug” that has a high affinity for the “right target”, administered at the “right time”.

The extracellular phase of neuron-to-neuron spread of pathological tau via transsynaptic propagation is a promising target for tau-directed therapy development [23,49]. Trials targeting the clearance of extracellular tau with humanized immunoglobulin-G4 antibodies that bind to the N-terminus of tau have been negative. This could be explained by the relatively low concentration of extracellular tau and the target epitope. The largest fraction of extracellular tau in the cerebrospinal fluid (CSF) is the mid-domain of tau, which is increased in AD compared with controls. There are now investigations of drugs targeting the mid-region epitope, with several anti-tau vaccines and antibodies in phase 2 or 3 clinical trials (Fig. 2) [49]. A phase 2a study of bepranemab, a monoclonal antibody targeting the tau mid-domain, showed no significant difference between active treatment and placebo on the primary endpoint (Clinical Dementia Rating Scale – Sum of Boxes [CDR-SB]), but a drug-placebo difference in favor of bapranemab was observed in a subset of key secondary endpoints, including rate of tau accumulation and change in the AD Assessment Scale-Cognitive Subscale [65]. Another monoclonal antibody targeting the tau microtubule-binding domain, etalanetug, is currently being investigated in a phase 2 and phase 2/3 trial in combination with lecanemab [66,67].

Inhibiting the production of tau intracellularly also appears to be a promising strategy. For example, BIIB080, an intrathecally administered antisense oligonucleotide that selectively targets *MAPT* messenger RNA to reduce the synthesis of tau protein, is currently being investigated in a phase 2 trial [68]. In a placebo-controlled, phase 1b trial of patients with early AD, exploratory analyses demonstrated that BIIB080 was associated with a dose-dependent and sustained reduction in soluble tau protein in CSF (phosphorylated tau [p-tau]−181 and total tau), and a reduction in aggregated parenchymal tau pathology on tau PET [69,70]. A numerical difference favoring high-dose BIIB080 groups compared with matched external controls was observed on multiple cognitive and functional clinical assessments [71].

Other promising targets include the reduction of tau phosphorylation (e.g., with phosphatase modifiers and kinase inhibitors), tau aggregation inhibitors, microtubule stabilizers, and tau degraders (Fig. 2) [49]. In addition, therapies targeting key PTMs, including acetylation inhibitors, deglycosylation inhibitors, and caspase inhibitors, are in development [49].

## 4 Role of tau biomarkers in the future patient journey

Tau biomarkers have a critical role in drug development and are expected to transition to clinical use [23]. The most established ways of measuring Aβ and tau levels in AD include PET and CSF. However, the recent emergence of blood-based biomarkers (BBMs) has the potential to transform the clinical care pathway with lower cost and improved accessibility and acceptability [72].

Tau aggregates can be visualized and quantified in vivo with tau PET [73,74]. Currently, there is one tau-PET tracer approved by the FDA and the European Medicines Agency available to assess tau burden in patients with AD ([18F]flortaucipir) [75]. Next-generation tau-PET tracers, such as [18F]MK6240, [18F]FRO-9484, [18]PI-2620, and [18F]GTP1 are not available for general clinical practice, but are currently used in research settings and clinical trials for AD and other tauopathies [76]. The value of tau PET has been demonstrated in a research setting; however, it is infrequently used in clinical practice due to limited access and a lack of reimbursement [77].

Tau PET is increasingly utilized in AD clinical trials to enrich or stratify patient populations according to their level of tau burden, and as an endpoint in trials of investigational Aβ- and tau-directed therapies [8,9,78,79]. This modality can provide important insights into clinical progression and disease mechanisms. Progression of tau pathology through the Braak stages is associated with the occurrence and progression of cognitive impairment [52]. Throughout the lifespan, tau pathology accumulates slowly in the MTL regions, becoming ubiquitous in older adults [80,81]. In the presence of Aβ, the proliferation of MTL-tau accelerates, spreading into the neocortex and correlating with worsening cognition. This event is thought to be a critical milestone in the AD pathogenic process, in a term coined by Keith Johnson as “caTAUstrophe” [[82], [83], [84]]. One recent study of 246 cognitively unimpaired older adults found that the timeline to this critical “caTAUstrophe” event was dependent on the level of existing age-related MTL-tau. When MTL-tau levels were high, rapid tau proliferation was observed at the first detectable signs of Aβ abnormalities, but in individuals with low MTL-tau levels, tau proliferation was delayed until Aβ was widespread [84]. These findings highlight the utility of tau PET, not only for the staging of AD severity, but also in predicting clinical outcomes.

In a phase 3 trial of the anti-Aβ monoclonal antibody donanemab, participants were recruited and stratified by quantifying the level of tau-PET tracer ([F18]flortaucipir) uptake; however, there are currently no standardized criteria for defining levels of tau burden. Patients with low levels of tau PET (defined as a standardized uptake value ratio [SUVr] <1.10) were excluded from the study, with a rationale that their expected rate of disease progression would not be sufficient to allow for reliable measurement of clinical decline or of study treatment effects within an 18-month study duration. In this study, Aβ-positive individuals with symptomatic AD who had low-to-medium levels of tau tracer uptake on PET (defined as SUVr >1.10 but ≤1.46 [*n* = 1182], with the upper threshold chosen to separate those in the top quartile of tau-PET level) experienced greater clinical benefit of the treatment compared with those with higher baseline tau-PET levels (>1.46 SUVr [*n* = 552]) [8]. In addition, in post-hoc analyses of the phase 3 trial of lecanemab, participants who had low levels of tau-PET tracer uptake ([F18]MK6240 radiotracer) at baseline (defined as SUVr <1.06 [*n* = 141]) demonstrated a low spread of tau in early Braak regions, with data supporting stability or improvement in CDR-SB in these individuals over the 18-month study period [85].​ These findings not only support the hypothesis that anti-Aβ monoclonal antibody therapy is most beneficial in the earliest stages of the disease, but also highlight the potential utility of tau PET as a tool to inform decisions regarding patients most likely to respond best to treatment.

There are several CSF and plasma tau biomarkers that can identify individuals with AD pathology; however, they have varying levels of specificity depending on the disease stage. Tau is phosphorylated at different positions at different times in the disease process, creating specific p-tau isoforms (e.g., p-tau181, p-tau217, and p-tau231) [6]. These p-tau isoforms become abnormal in the preclinical and MCI stages of AD around the same time that Aβ PET becomes abnormal and before significant changes are detectable on tau PET [6,[86], [87], [88], [89]]. P-tau is considered a downstream product of Aβ-induced pathological processes whereby Aβ triggers the secretion and phosphorylation of tau. Studies suggest that p-tau231 increases early, followed by p-tau217 and then p-tau181 [89,90]. Consequently, p-tau biomarkers are strongly associated with increasing AD pathology, with some (p-tau181 and p-tau217) showing strong correlations with both Aβ plaque burden and tau NFTs [91]. Some plasma p-tau assays measuring p-tau217 can predict the presence of Aβ pathology with accuracy comparable to PET and CSF testing [[91], [92], [93]]. Plasma p-tau217 has been shown to increase steadily throughout the course of AD progression [94,95]; however, studies have observed only a modest performance of plasma p-tau217 as a staging biomarker, suggesting that it is not sufficiently accurate to robustly estimate continuous tau-PET burden [96,97]. Tau microtubule-binding region tau species containing residue 243 (MTBR-tau243), measured in the CSF, have been found to be more specific for insoluble tau aggregate pathology, potentially better reflecting tau NFT pathology in AD. MTBR-tau243 becomes abnormal later in the disease continuum and shows a strong correlation with tau PET and disease progression [98]; the ability of CSF MTBR-tau243 to predict tau-PET positivity (based on [18F]flortaucipir SUVr quantification) and clinical outcomes is further improved when combined with p-tau205. To improve acceptability and to enable scaling of the MTBR-tau243 biomarker, a plasma-based assay of endogenously cleaved MTBR-tau243 (eMTBR-tau243) has been developed. Plasma eMTBR-tau243 has shown strong associations with tau-PET binding and cognitive performance in a large clinical cohort [99]. There are also potential biomarkers of aggregation-relevant phosphorylation sites evident in pre-tangle species of tau, p-tau serine 262 and 356, which may pave the way for the quantification of early-stage soluble (prefibrillar) tau assemblies in CSF that may not be detectable using tau PET [100].

In summary, over the past decade, studies have indicated the potential utility of tau biomarkers in a diagnostic role, exhibiting predictive capacity, determining optimal treatment windows, and providing prognostic insights [84]. While (e)MTBR-tau243 and p-tau serine 262 and 356 have promise as biomarkers for disease staging and may be potential future alternatives to tau PET, these biomarkers are in the early stages of their development, and additional data are required to understand their utility in reflecting the presence and dynamics of intracellular NFTs. The context of use of the tau biomarker armamentarium will need to be carefully considered within trial and clinical practice settings. With the potential advent of novel classes of DTTs, including those directed toward tau, a comprehensive individual clinical biomarker profile will likely be needed.

## 5 The evolving AD patient journey

The recent emergence of DTTs for AD has led to a paradigm shift in the concept of the ideal AD patient journey. Currently, the only class of DTTs available clinically, anti-Aβ monoclonal antibodies, are indicated for the early symptomatic stages of the disease and require confirmation of amyloid pathology. Preliminary data suggest that there is a greater clinical benefit the earlier in the disease course they are initiated [8,9]. This has led to a shift from a clinical care model focused on symptom management and late-stage diagnosis, to a patient journey centered around an urgency to achieve a biomarker-confirmed diagnosis of AD in the earliest clinical stages [101]. However, while our conceptualization has shifted, the implementation of changes in the patient journey is still in its infancy and faces numerous complex challenges spanning capacity, resources, and educational needs [101]. Patients, family members, clinicians, and healthcare systems are all impacted by the transition to earlier detection and diagnosis of cognitive impairment. The potential advent of an additional class of DTTs for AD may further accelerate this shift, increasing the number of patients seeking earlier evaluation, and heightening the urgency to address existing challenges. Additionally, a tau-directed therapy would require workflow changes in terms of treatment decision-making, administration, and monitoring. Finally, the potential for treatments to target different stages of disease will necessitate an approach that requires biomarker-led, patient-focused discussions on which treatments are optimal when.

### 5.1 Treatment decision-making

As alternative classes of DTTs become available, treatment decision-making will inform a more personalized approach to care. However, this will also lead to additional complexity in treatment decision-making, particularly in terms of the type of treatment, the timing of treatment initiation, and the benefits and risks of potential combination or sequential therapy regimens. This will also increase the need for shared decision-making between healthcare professionals and individual patients.

**Fig. 3:**
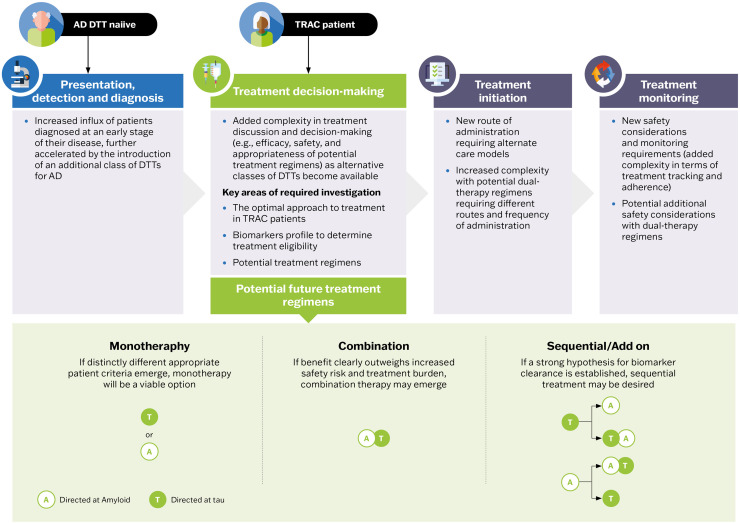
Shifts in the future AD patient journey, with the advent of a tau-directed DTT. Predicted key shifts driven by a potential greater focus on tau in the future AD patient journey are highlighted at each stage. Abbreviations: A, amyloid; AD, Alzheimer’s disease; DTT, disease-targeted treatment; T, tau; TRAC, treatment-related amyloid clearance.

Clinicians will likely build a comprehensive clinical picture, including an individual biomarker profile, to determine which drug and treatment regimen would be the most appropriate. An overview of the ways in which tau therapeutics could potentially shape the future AD patient journey is illustrated in Fig. 3. Currently, it is unknown whether a future tau-directed therapy would be used as a monotherapy, in combination with anti-Aβ monoclonal antibody therapy, or in sequential or add-on treatment approaches. If distinctly different appropriate patient criteria emerge for Aβ-directed and tau-directed therapies, there may be instances where monotherapy would be a viable option. However, given the multifactorial nature of AD, the need for combination therapies or sequential treatment strategies is anticipated. Robust clinical trial data will be required to elucidate the benefit-risk profile of any potential treatment regimen, and to enable data-driven treatment decisions to be made. Another key gap in the existing evidence is the effects of existing and future DTTs in patients with AD and co-pathologies, which is the rule rather than the exception. Patients with AD may harbor a range of comorbid pathologies, including alpha-synuclein pathology, TAR DNA-binding protein-43 aggregation (TDP-43), and vascular changes [102,103]. Importantly, the presence of additional pathologies accelerates both the biological and clinical disease progression in AD [104,105]. Therefore, the existence of co-pathologies may influence the treatment choice that best addresses the most important pathologies for individual patients. Additionally, current treatment decisions are primarily made in the context of those who have not yet received DTTs. However, as alternative classes of DTTs become available, there will be numerous scenarios to navigate. For example, most individuals treated with anti-Aβ monoclonal antibodies demonstrate a negative visual read on an Aβ PET scan following Aβ plaque clearance, with many also showing declines in CSF and plasma p-tau biomarkers [9]. This state has recently been termed “treatment-related amyloid clearance”, or “TRAC”, by an Alzheimer’s Association-convened workgroup [6]. Therefore, treatment decisions regarding add-on or sequential DTTs in these individuals must also consider that DTTs will alter their clinical biomarker profile. The optimal approach to treatment in these cases, as well as decisions around the placement of tau-directed therapies in the future treatment landscape, will be data-driven following clinical and real-world investigations. The emergence of additional classes of DTTs for AD treatment will bring added complexity to treatment discussions and decision-making, in terms of the efficacy, safety, and appropriateness of potential treatment regimens.

### 5.2 Treatment administration and monitoring

New classes of DTTs may have unique routes of administration compared with anti-Aβ-monoclonal antibodies. Anti-Aβ-monoclonal antibodies currently require intravenous infusions every 4 or 2 weeks, with a weekly subcutaneous injection available for lecanemab following 18 months of treatment (an administration route that could potentially become available for use at treatment initiation in the future) [106]. Some investigational tau-directed therapies employ an intrathecal administration route [69]. Based on early clinical data, some intrathecal tau-directed therapies may require a lower dosing frequency (e.g., every 3 to 6 months) than that of currently available anti-Aβ-monoclonal antibody regimens [68,69]. A new class of DTT will have unique risk profiles and safety considerations, and likely have specific safety and monitoring requirements. Safety monitoring for anti-Aβ-monoclonal antibodies includes MRIs to monitor for Aβ-related imaging abnormalities (ARIA), a potential side effect of this drug class [107]. Appropriate adjustments will be required for clinical workflows in anticipation of the unique administration and monitoring needs of a second class of DTT. With the potential for dual-therapy regimens, differences in route and frequency of administration, along with additional monitoring protocols, may add complexity to treatment tracking and adherence. Moreover, additional safety considerations may arise with potential dual-therapy regimens requiring tailored care.

An additional consideration will be the monitoring of treatment effect if two classes of DTT are available that target different pathologies. This comes with the added complexity of interpreting biomarkers in the setting of multiple DTT use. Since DTTs alter the relationships between biomarkers evident in the natural history of the disease, the interpretation of biomarkers for diagnosis, staging, prognosis, and treatment eligibility will require adjustment following treatment or the introduction of combination or add-on therapies [6]. Criteria for when or if it is appropriate to stop a tau-targeting therapy based on specific clinical progression and/or biomarker results will be important to develop. The role of biomarkers in these scenarios remains to be elucidated and requires data-driven discussions following real-world investigation.

## 6 Conclusions

This review highlights the potential of tau as both a promising therapeutic target and as a valuable biomarker. AD is a multifactorial disease, and findings suggest that addressing multiple pathological drivers will be required to maximize clinical effect. Tau is a critical player in the pathophysiology of AD. It correlates more strongly with the severity and spread of symptomatology than Aβ plaque deposition, has a stronger association with future cognitive stage than Aβ plaque deposition or MRI markers, and can predict the response to anti-Aβ monoclonal antibodies. There are now several tau-directed agents, each with differing modes of action, in various stages of clinical development. Tau-based biomarkers are expanding our understanding of the AD pathological process and are beginning to play a greater role in a clinical research setting. Biomarkers that more closely correlate with NFTs add substantially to the tau biomarker toolkit. The potential advent of an effective tau-directed DTT for AD may increase the volume of patients presenting earlier in the disease continuum, and increase complexity in workflow, infrastructure, treatment decision-making, treatment administration and monitoring, and patient tracking. It could also provide new opportunities to personalize treatment and empower patients and their physicians with treatment choices.

## Funding

This work was supported by Biogen. The sponsor was involved in the review and approval of the manuscript.

## Disclosures

The authors declare the following financial interests/personal relationships that may be considered as potential competing interests:

Catherine J. Mummery has provided consultancy to Alector, Biogen, Eisai, Ionis, Lilly, MSD, Neurimmune, Novartis, Prevail, Roche/Genentech, and Wave. She is an advisor to the Drug Delivery Foundation. She has received honoraria for teaching and education from Biogen, Eisai, and Lilly, and received academic funding award from Biogen. She is supported by the NIHR UCLH Biomedical Research Centre.

Christopher Chen Li-Hsian declares no conflicts of interest.

Cristian A. Lasagna-Reeves is a shareholder of Monument Biosciences, Inc.

Rik Ossenkoppele has received research funding/support from Avid Radiopharmaceuticals, Janssen Research & Development, Roche, Quanterix, and Optina Diagnostics. He has given lectures in symposia sponsored by GE Healthcare, received speaker fees from Springer, is an advisory board/steering committee member for Asceneuron, Biogen, and Bristol Myers Squibb. All the aforementioned has been paid to his institutions.

Christopher C. Rowe provided consultation for Biogen, Eisai, Lilly, Roche, Cerveau Technologies, Enigma Biomedical, Merck, Novo Nordisk, and Prothena, and received grants paid to his institution from Biogen, Eisai, Roche, Novo Nordisk, Cerveau, and Enigma. He is supported by Austin Health and the National Health and Medical Research Council of Australia.

Douglas W. Scharre provided consultation to BrainTest, Eisai, Lilly, Biogen, and Otsuka, and has received grants paid to his institution from Roche, Precision Medicine, Avanir, Premier Research Group, Genetech, Cervel Therapeutics, UCB Biopharma, Hanssen, Vivoryon Therapeutics, Cassava, BioVie, uniQure, Cognition Therapeutics, Cognitive Research Corporation, Biogen, EIP, and Cognito.

Huali Wang provided consultation to Biogen, Eisai, Lilly, and Lundbeck pharmaceuticals companies. She is supported by a grant from the Science and Technology Innovation 2030- Major Project (2021ZD0201805).

Simon Kyaga is an employee and shareholder of Biogen International GmbH, Neuhofstrasse, Baar, Switzerland.

Jeffrey L. Cummings has provided consultation to Acadia, Acumen, ALZpath, Annovis, Aprinoia, Artery, Axsome, Biogen, Biohaven, BioXcel, Bristol-Myers Squib, Cervomed, Eisai, Fosun, GAP Foundation, Green Valley, Hummingbird Diagnostics, IGC, Janssen, Kinoxis, Lighthouse, Lilly, Lundbeck, LSP/eqt, Mangrove Therapeutics, Merck, MoCA Cognition, New Amsterdam, Novo Nordisk, NSC Therapeutics, Optoceutics, Otsuka, Oxford Brain Diagnostics, Praxis, Prothena, ReMYND, Roche, Scottish Brain Sciences, Signant Health, Simcere, sinaptica, T-Neuro, TrueBinding, and Vaxxinity pharmaceutical, assessment, and investment companies. He is supported by NIGMS grant P20GM109025, NIA R35AG71476, NIA R25AG083721–01, NINDS RO1NS139383, Alzheimer’s Disease Drug Discovery Foundation (ADDF), Ted and Maria Quirk Endowment, and Joy Chambers-Grundy Endowment.

## CRediT authorship contribution statement

**Catherine J. Mummery:** Writing – review & editing, Conceptualization. **Christopher Chen Li-Hsian:** Writing – review & editing, Conceptualization. **Cristian A. Lasagna-Reeves:** Writing – review & editing, Conceptualization. **Rik Ossenkoppele:** Writing – review & editing, Conceptualization. **Christopher C. Rowe:** Writing – review & editing, Conceptualization. **Douglas W. Scharre:** Writing – review & editing, Conceptualization. **Huali Wang:** Writing – review & editing, Conceptualization. **Simon Kyaga:** Writing – review & editing, Conceptualization. **Jeffrey L. Cummings:** Writing – review & editing, Conceptualization.

## Declaration of competing interest

The authors declare the following financial interests/personal relationships which may be considered as potential competing interests:

This work was supported by Biogen. The sponsor was involved in the review and approval of the manuscript. The authors declare the following financial interests/personal relationships that may be considered as potential competing interests: Catherine J. Mummery has provided consultancy to Alector, Biogen, Eisai, Ionis, Lilly, MSD, Neurimmune, Novartis, Prevail, Roche/Genentech, and Wave. She is an advisor to the Drug Delivery Foundation. She has received honoraria for teaching and education from Biogen, Eisai, and Lilly, and received academic funding award from Biogen. She is supported by the NIHR UCLH Biomedical Research Centre. Christopher Chen Li-Hsian declares no conflicts of interest. Cristian A. Lasagna-Reeves is a shareholder of Monument Biosciences, Inc. Rik Ossenkoppele has received research funding/support from Avid Radiopharmaceuticals, Janssen Research & Development, Roche, Quanterix, and Optina Diagnostics. He has given lectures in symposia sponsored by GE Healthcare, received speaker fees from Springer, is an advisory board/steering committee member for Asceneuron, Biogen, and Bristol Myers Squibb. All the aforementioned has been paid to his institutions. Christopher C. Rowe provided consultation for Biogen, Eisai, Lilly, Roche, Cerveau Technologies, Enigma Biomedical, Merck, Novo Nordisk, and Prothena, and received grants paid to his institution from Biogen, Eisai, Roche, Novo Nordisk, Cerveau, and Enigma. He is supported by Austin Health and the National Health and Medical Research Council of Australia. Douglas W. Scharre provided consultation to BrainTest, Eisai, Lilly, Biogen, and Otsuka, and has received grants paid to his institution from Roche, Precision Medicine, Avanir, Premier Research Group, Genetech, Cervel Therapeutics, UCB Biopharma, Hanssen, Vivoryon Therapeutics, Cassava, BioVie, uniQure, Cognition Therapeutics, Cognitive Research Corporation, Biogen, EIP, and Cognito. Huali Wang provided consultation to Biogen, Eisai, Lilly, and Lundbeck pharmaceuticals companies. She is supported by a grant from the Science and Technology Innovation 2030- Major Project (2021ZD0201805). Simon Kyaga is an employee and shareholder of Biogen International GmbH, Neuhofstrasse, Baar, Switzerland. Jeffrey L. Cummings has provided consultation to Acadia, Acumen, ALZpath, Annovis, Aprinoia, Artery, Axsome, Biogen, Biohaven, BioXcel, Bristol-Myers Squib, Cervomed, Eisai, Fosun, GAP Foundation, Green Valley, Hummingbird Diagnostics, IGC, Janssen, Kinoxis, Lighthouse, Lilly, Lundbeck, LSP/eqt, Mangrove Therapeutics, Merck, MoCA Cognition, New Amsterdam, Novo Nordisk, NSC Therapeutics, Optoceutics, Otsuka, Oxford Brain Diagnostics, Praxis, Prothena, ReMYND, Roche, Scottish Brain Sciences, Signant Health, Simcere, sinaptica, T-Neuro, TrueBinding, and Vaxxinity pharmaceutical, assessment, and investment companies. He is supported by NIGMS grant P20GM109025, NIA R35AG71476, NIA R25AG083721–01, NINDS RO1NS139383, Alzheimer’s Disease Drug Discovery Foundation (ADDF), Ted and Maria Quirk Endowment, and Joy Chambers-Grundy Endowment.

If there are other authors, they declare that they have no known competing financial interests or personal relationships that could have appeared to influence the work reported in this paper.
